# Preliminary Findings in Cataract Surgery Using a Digital Microscope Platform With Infrared Illumination

**DOI:** 10.1155/joph/6715584

**Published:** 2026-05-19

**Authors:** Brady Wortz, Thiago Moulin, Mariam Bakhtyari, Renee C. Bondurant, Robert J. Weinstock

**Affiliations:** ^1^ Department of Ophthalmology, The Eye Institute of West Florida, Largo, Florida, USA

**Keywords:** cataract surgery, infrared microscope, phacoemulsification

## Abstract

**Purpose:**

To evaluate the safety and efficacy of infrared (IR) illumination using a digital microscope platform to perform uncomplicated cataract surgery.

**Methods:**

A retrospective case series of 39 eyes (33 patients) that underwent cataract surgery under topical anesthesia. Patients were asked to choose between traditional microscope and IR microscope illumination prior to surgery. All cataract procedures were performed by a single surgeon. Primary outcomes were intraoperative complications, surgical time, effective phacoemulsification time (EPT), and patient preferences.

**Results:**

The mean patient age was 74.1 ± 9.24 years, with 35.9% male eyes, and 20.5% underwent femtosecond laser‐assisted surgery. All patients in this series preferred the IR microscope illumination. Mean surgical time was 8.33 ± 4.02 min, and mean EPT was 1.77 ± 0.84 s. No intraoperative complications were observed.

**Conclusions:**

Cataract surgery using IR illumination demonstrated excellent safety and efficacy; patients preferred the IR illumination over a traditional microscope illumination. These preliminary findings support further investigation of IR‐based visualization technology in ophthalmic surgeries.

## 1. Introduction

Cataract surgery is typically performed using a high‐intensity white‐light microscope, which, while essential for visualization, can cause significant discomfort for patients under topical anesthesia [1–4]. Bright surgical illumination has been linked to increased intraoperative light sensitivity and pain and can cause phototoxic maculopathy due to prolonged retinal exposure [5]. Additionally, the need to visualize transparent structures such as the anterior lens capsule can be particularly challenging in mature cataracts with a poor red reflex, often necessitating the use of capsular dyes that can add procedural steps and carry a small risk of intraocular toxicity while also increasing the amount of time of microscope illumination [6].

Infrared (IR) illumination offers a novel alternative to traditional white‐light microscopes. IR wavelengths (850–1300 nm) are invisible to the human eye, which may decrease or eliminate intraoperative photosensitivity and discomfort. An early study suggested phacoemulsification could be completed under IR guidance and noted greater patient satisfaction and faster visual recovery [7]. Furthermore, IR imaging may enhance the contrast of transparent ocular tissues (such as the anterior capsule) even in dense cataracts, which may reduce or eliminate the surgeon’s need for staining dyes.

This retrospective case series evaluates patient preferences for the use of a novel IR illumination microscope during cataract surgery to determine efficacy and safety of the microscope.

## 2. Materials and Methods

### 2.1. Study Design and Population

This retrospective study evaluated the records of patients who underwent cataract surgery at the Eye Institute of West Florida’s Largo’s Ambulatory Surgery Center between February 11, 2025, and March 4, 2025. Patient records were excluded if they had undergone complex cataract surgery. A total of five surgeries were combined with the insertion of the iStent Infinite (Glaukos) for the treatment of concurrent elevated intraocular pressure. This study was conducted according to the principles of the Declaration of Helsinki, Institutional Review Board approval was granted by WCG (Cary, NC), and informed consent was provided by all patients.

### 2.2. Surgical Technique

All patients were queried about their preference in microscope light (being shown both a traditional white‐light microscope light and an IR microscope light) immediately before surgery started. Patients were asked about comfort levels with each option, and whichever microscope light the patient preferred was used during surgery.

All procedures were performed under topical anesthesia by the same experienced surgeon (Robert J. Weinstock). Patients either underwent cataract surgery with or without the aid of a femtosecond laser pretreatment, which included capsulotomy creation and crystalline lens fragmentation. Cataract surgery was initiated by two 1.6‐mm‐wide clear corneal incisions 70° apart, performed using a diamond keratome, followed by injection of a cohesive viscoelastic device to fill the anterior chamber. Microcapsulorrhexis forceps were then used to perform capsulorrhexis or to remove the capsulotomy button in laser‐pretreated eyes, followed by hydrodissection and crystalline lens rotation with a 27‐gauge cannula. The Stellaris Elite system (Bausch and Lomb) was used to perform bimanual phacoemulsification and irrigation/aspiration of the crystalline lens and cortex, respectively. All eyes were implanted with the Akreos MICS M160L IOL (Bausch + Lomb). All patients underwent the same postoperative treatment comprising a combination of an antibiotic, steroid, and nonsteroidal anti‐inflammatory drop.

### 2.3. IR Microscope

Beyeonics One (Beyeonics) is a fully digital surgical microscope platform. The system replaces conventional optical microscopes with a virtual reality headset. The device was designed to improve functionality and ergonomics by allowing the surgeon to adjust lighting, digital zoom, operating modes, and more with head gestures. The system provides coaxial illumination in visible, IR, or combined modes and is the first commercially available ophthalmic surgical microscope that qualifies as a Group 1 device under ANSI Z80.36 (which indicates no potential light hazard).

**FIGURE 1:**
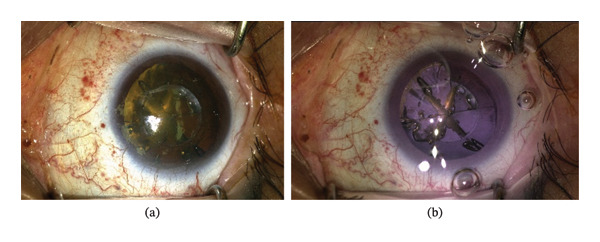
(a) An eye as viewed by the surgeon with a traditional white‐light microscope. (b) An eye as viewed by the surgeon with an infrared illumination microscope.

This microscope exhibits the IR light as a purple/pinkish color to the surgeon. Figure 1(a) shows illumination under standard microscope white light, while Figure 1(b) shows illumination with the IR microscope. The greater tissue penetration and higher retinal reflectance of the IR light provides the surgeon with better visualization and depth perception through any density of cataract. The IR microscope was designed to substantially decrease or eliminate phototoxicity to the retina, which may potentially improve patient comfort during surgery and visual recovery.

### 2.4. Outcome Measures

The primary outcome measures were the number of intraoperative complications when using the IR microscope, surgical time (“cut to close”), and effective phaco time (EPT). Secondary outcomes included patient preferences for the microscope.

## 3. Results

A total of 33 patient records (*n* = 39 eyes) met the inclusion criteria. All patients (*n* = 33, 100%) indicated a preference for the IR microscope compared to the traditional white‐light microscope. Mean age was 74 ± 9.24 years, 14 patients (42.4%) were male, and 8 eyes (20.5%) underwent femtosecond laser‐assisted surgery. There were no intraoperative complications observed. The mean surgical time was 8.33 ± 4.02 min, and mean EPT was 1.77 ± 0.84.

## 4. Discussion

The findings from our retrospective case series show that using IR illumination during cataract surgery is both safe and efficient. There were no intraoperative or postoperative complications in any eye, and the mean surgical time (8.33 min) and low EPT (1.77 s) indicate that the system can achieve excellent efficacy comparable to conventional microscopes. These findings align with other early experiences using head‐mounted digital visualization in ophthalmology. For example, Sorkin et al. reported on 60 cataract cases performed with a similar 3D head‐mounted display system and found it could be easily integrated into routine surgery with no adverse events, while providing high image quality and ergonomic benefits for the surgeon [8]. Likewise, in both corneal and vitreoretinal surgeries, the same platform as used in this case series was used without complications or any noticeable lag in the real‐time video feed [9, 10], underlining the feasibility of fully digital, heads‐up visualization across ophthalmic procedures. Our series adds to this growing evidence by finding a low EPT and an expected mean surgical time, while also including patient preferences for the IR illumination.

One advantage of this type of microscope system is the use of IR illumination in addition to or in lieu of visible light used in traditional microscopes that helps facilitate visualization of the anterior capsule and the different tissue planes of the lens itself and of cortex during irrigation/aspiration, among other structures [11]. Delaney‐Gesing reported a substantial decrease in the need for staining dyes used in capsular staining during challenging cases (e.g., dense cataracts, vitreous hemorrhage, and cloudy corneas), streamlining the surgical procedure [11].

An IR‐based surgical microscope offers potential benefits for patient safety through faster visual recovery and less light toxicity [7]. Because IR light is outside the visible spectrum, the patient perceives little to no light during surgery. In contrast, traditional microscopes operate in the visible light spectrum, and studies have shown patients find the bright light uncomfortable or dazzling and have been implicated in retinal photic injuries even during relatively short cataract cases [1–5, 7, 12]. The authors believe the exclusive use of IR during surgery may eliminate these complications. This benefit is especially relevant for long or complex surgeries, where extended light exposure would otherwise compound the risk of retinal phototoxicity. Another consequence of avoiding intense visible light is faster visual recovery in the immediate postoperative period. Bright surgical lights can “bleach” photoreceptors, leading patients to experience washed‐out vision or color tints for minutes to hours after surgery [13–15]. In one of the first studies that evaluated IR cataract surgery, Kim reported that patients recovered vision more quickly and did not report the transient visual field defects (such as pink or yellow tinges) that were noted by patients who underwent standard illumination [7]. Similarly, Eom et al. [16] reported mean patient suffering was significantly smaller and patient cooperation was significantly greater when patients underwent cataract surgery with an illuminated chopper compared to a conventional chopper, noting the illuminated device produced less glare and anxiety without increasing operating time or damaging the corneal endothelium. Finally, in cases of bilateral, same‐day surgery, the potentially faster visual recovery post‐IR use could reduce the time patients need to have clear vision return.

There are also surgeon ergonomics that may benefit from the use of this IR microscope. Traditional analog microscopes provide superb optical resolution and true‐color visualization, but they tether the surgeon’s head to the eyepiece in an often‐strained forward posture. Chronic neck and back pain are well‐recognized occupational hazards among ophthalmic surgeons. Waisberg et al. found a majority of surgeons reported musculoskeletal pain attributed to long hours at the microscope [17]. A head‐mounted display (used in this IR microscope) can reduce strain by enabling the surgeon to look straight ahead at a virtual screen, positioning his/her body in the most comfortable position for surgery, regardless of the position of a screen or microscope oculars [8]. In our series, the primary surgeon subjectively reported less fatigue, which echoes the improved ergonomics noted in other digital microscope studies [17].

This study is not without its limitations, however, including its retrospective nature, small number of records included, no formal patient preference analysis, and no follow‐up beyond typical clinic standards. The use of femtosecond laser‐assisted cataract surgery techniques may alter surgical time, which might have had a minor bias in outcome measures although we did not compare the femtosecond outcomes to the non‐femtosecond outcomes.

However, we believe these limitations are more than offset by the strengths of the review, which reinforces the safety and efficacy of an IR microscope used in cataract surgery. When compared to traditional microscopes, the digital IR system provides an ergonomically superior working environment for the surgeon and augments his/her surgical view with advanced technology while maintaining image clarity and depth perception. Future studies should be prospective in nature and directly compare outcomes with an IR microscope to a traditional white‐light microscope, both in patient/surgeon comfort and in objective outcomes.

## 5. Conclusions

This case series found an IR‐based digital microscope provided excellent safety and efficacy and was overwhelmingly preferred by patients compared to a traditional white‐light microscope.

## Funding

This study was supported by a grant from Beyeonics, Haifa, Israel.

## Conflicts of Interest

Robert J. Weinstock is a consultant for Beyeonics. The other authors declare no conflicts of interest.

## Data Availability

The data that support the findings of this study are available from the corresponding author upon reasonable request.
